# Bovine Gammaherpesvirus 6 Tropism in the Natural Host

**DOI:** 10.3390/v16111730

**Published:** 2024-11-03

**Authors:** Rosalie Fabian, Giuliana Rosato, James P. Stewart, Anja Kipar

**Affiliations:** 1 Institute of Veterinary Pathology, Vetsuisse Faculty, University of Zurich, 8057 Zurich, Switzerland; rosalie.fabian@uzh.ch (R.F.); giuliana.rosato@uzh.ch (G.R.); 2 Department of Infection Biology & Microbiomes, University of Liverpool, Liverpool L3 3RF, UK; j.p.stewart@liverpool.ac.uk

**Keywords:** bovine herpesvirus-6, gammaherpesvirus, cattle, target cells

## Abstract

Bovine gammaherpesvirus 6 (BoHV-6) is endemic in cattle in Europe, with a high prevalence. There is evidence that the virus is a commensal and not associated with disease processes. For other gammaherpesviruses, it is known that they have a rather specific target cell spectrum, generally including B cells and, at least in the early phase of infection, the epithelium of the respiratory tract. In a previous study we detected BoHV-6 by quantitative PCR for the gB gene sequence of BoHV-6 in lung, bronchial lymph nodes, spleen and tongue with variable loads, suggesting cells in these tissues as target cells. In the present study, formalin-fixed, paraffin embedded samples of the same tissues from 10 cattle, with high overall BoHV-6 copy numbers, were examined by RNA in situ hybridization for BoHV-6 ORF73. This revealed extremely limited viral ORF73 transcription. A signal was only detected in individual lymphocytes within lymphatic follicles in bronchial lymph nodes, and within very rare alveolar epithelial cells and interstitial cells in the lungs, without any evidence of pathological changes in the tissues. No signal was detected in the spleen or in the oral mucosa of the tongue. The results are consistent with previous findings with other gammaherpesviruses, murine herpesvirus-68, ovine herpesvirus-2 and/or Epstein–Barr virus. They provide further evidence that BoHV-6 is without any consequence to the host and can indeed represent a commensal in cattle.

## 1. Introduction

Bovine gammaherpesvirus 6 (BoHV-6; formally *Macavirus bovinegamma*6), first described in 1998 [1], is a member of the herpesvirus subfamily of Gammaherpesvirinae [2], among which are several viruses of veterinary and medical relevance, including malignant catarrhal fever (MCF) viruses, such as ovine herpesvirus-2 (OvHV-2; formally *Macavirus ovinegamma*2) and alcelaphine herpesvirus-1 (AlHV-1; formally *Macavirus alcelaphinegamma*1), Epstein–Barr virus (EBV; *Lymphocryptovirus humangamma*4) and murine herpesvirus-68 (MHV-68; formally *Rhadinovirus muridgamma*4) [3].

Studies in the USA and Europe have shown that BoHV-6 is endemic in cattle, with an overall prevalence of over 30% [1,4,5,6]. Infection has also been reported in water buffaloes (*Bubalus bubalis*) in Brazil [7] and bison in the USA [5], which indicates that the natural host range goes beyond cattle. A recent study has shown that infection can be detected in cattle as early as 1 day after birth but that the prevalence of BoHV-6 infection in cattle increases with age [4]. The same study found no evidence that BoHV-6 is transmitted transplacentally, indicating that perinatal infection or those shortly after birth are likely [4]. However, a subsequent study from Brazil then detected the virus by nested PCR (nPCR) in 77% of aborted fetuses with confirmed *Histophilus somni* infection. The virus was identified in multiple organs including spleen, myocardium, kidney and brain. In addition to being co-infected with *H. somni*, 60% of the BoHV-6 positive fetuses showed triple and quadruple infections with other pathogens, such as *Brucella abortus*, bovine viral diarrhea virus, bovine alphaherpesvirus 1 and *Neospora caninum* [8]. There is also an earlier report that described a case of an aborted fetus in Canada in which BoHV-6 was found in brain and lymph nodes [9]; this fetus was also found to be infected with *Neospora caninum*. This protozoan parasite leads to abortion in the earlier phase of pregnancy, inducing placental epithelial necrosis, serum leakage and maternal mononuclear cell infiltration [10]. Destruction of the placental barrier could therefore have allowed for BoHV-6 to spread into fetal tissue, provided the mother was viremic. Several studies have suggested an association of BoHV-6 with specific diseases, such as lymphoproliferative, reproductive and/or respiratory disease, but these generally detected co-infections with other infectious agents [8,9,11,12,13,14,15]. Evidence of a direct pathogenic effect of BoHV-6 is restricted to a single aborted bovine fetus co-infected with *Histophilus somni,* which histologically exhibited necrotizing myocarditis where BoHV-6 was the only agent detected in the cardiac tissue through PCR, and interstitial pneumonia in a fetus and a cow in which no other pathogen was detected [14,15]. In contrast, a recent study by our group on a large cohort of cattle in Europe did not find evidence that BoHV-6 is associated with specific diseases in cattle [4]. Overall, BoHV-6 appears to be a commensal rather than a pathogen in cattle.

Experimental studies using MHV-68 and OvHV-2 in their natural hosts have suggested that some gammaherpesviruses initially replicate in the lungs, in alveolar and respiratory epithelial cells, respectively, and then establish latency in lymphoid tissues [16,17]. First evidence of the sites of BoHV-6 infection was obtained in a recent study where a quantitative PCR detecting the glycoprotein B (gB) gene sequence of BoHV-6 detected viral genome in lungs, tongue, bronchial lymph nodes and spleen, suggesting that systemic spread of the virus is common, and that the virus might also be shed orally [4].

It is known for several Gammaherpesvirinae that they have a narrow cell tropism in their natural hosts. They generally infect lymphocytes and epithelial cells [16,17,18], and some, like Kaposi’s sarcoma-associated herpesvirus (KSH) and EBV, have a tropism for endothelial cells [19], while another relative, MHV-68, infects macrophages [20]. Gammaherpesviruses establish latency in B cells; some also cause persistence-associated tumor formation [16,17,21]. Considering the PCR-based results of our recent study [4], it appears possible that BoHV-6 infects respiratory, alveolar and squamous epithelial cells as well as lymphocytes.

The aim of the present study was to identify the viral target cells by in situ examination of tissues found to carry the virus. Knowledge on the cellular tropism of BoHV-6 would help to better understand how the virus is shed and whether it has any, even subtle, pathogenic effect.

## 2. Materials and Methods

### 2.1. Animals and Tissue Samples

In a previous study, our group screened a large cohort of healthy slaughtered, and clinically diseased cattle without MCF that had been subjected to a diagnostic post-mortem examination for infection with a range of gammaherpesviruses known to affect ruminants, including ovine herpesvirus-1 (OvHV-1), OvHV-2, BoHV-6, bison lymphotropic herpesvirus (LHV) and caprine herpesvirus-2 (CpHV-2), by quantitative PCR [4]. Animals originating from different European countries, Switzerland, the UK, Finland, Belgium and Germany, and were of various breeds. In all animals, lung, bronchial lymph node (BLN), spleen and tongue (mainly the epithelium) were examined. These tissues were selected because they had previously been shown to be infected by the viruses [22,23]. BoHV-6 was the only gammaherpesvirus detected in these animals, with an overall prevalence of 32%. The quantitative PCR used TaqMan primers and probe directed against the gB gene sequence of BoHV-6 [4,6]. In addition to the samples tested by PCR, samples from the same tissues had been collected after slaughter or during the diagnostic post-mortem examination for histological and further in situ examinations. These were fixed in 10% buffered formalin for 48 h.

### 2.2. Tissue Processing, Histology and RNA In Situ Hybridization (RNA-ISH)

After fixation, the tissue samples were either trimmed directly or after storage in 70% ethanol for shipping until further processing. After trimming, the tissue specimens were routinely paraffin wax-embedded for histological examination and RNA-ISH.

Tissues from the 10 BoHV-6-infected cattle with the highest copy numbers in (some of) the tissues (up to 21,593 copies/100 ng DNA), were selected for the present study (Table 1). A first section (3 µm) was prepared and routinely stained with hematoxylin and eosin (HE) and histologically examined to assess the general preservation of the tissue and to identify any pathological processes. This excluded one BLN from any further examinations due to poor tissue preservation (Table 1).

From all other tissues, consecutive sections (4 µm) were prepared and tested with an RNAscope^®^ oligoprobe for *Bos taurus* peptidylprolyl isomerase B (PPIB) to confirm RNA preservation and quality, following the manufacturer’s (Advanced Cell Diagnostics, Newark, NJ, USA) protocol. These yielded good PPIB signals (Supplemental Figure S1); hence, consecutive sections were then subjected to RNA-ISH for BoHV-6 ORF73 (isolate Pennsylvania 47; Genbank NC_024303.1), a putative immediate early protein of BoHV-6 [2], using RNAscope^®^ oligoprobes. An automated RNAscope 2.5 Detection Reagent Kit (Brown) was used according to the manufacturer’s (Advanced Cell Diagnostics) as well as a previously published protocol [24], with slight adjustments. Briefly, sections were heated to 60 °C for 1 h and subsequently deparaffinized. Permeabilization was achieved by incubating the section in pretreatment solution 1 (RNAscope^®^ Hydrogen Peroxide) for 10 min at room temperature (RT), followed by boiling in RNAscope^®^ 1× Target Retrieval Reagent solution at 100–104 °C for 25 min in a pressure cooker and washing in distilled water and ethanol. After digestion with RNAscope^®^ Protease Plus for 30 min at 40 °C, sections were hybridized with the oligoprobes at 40 °C in a humidity control tray for 2 h (HybEZTM Oven, ACD Advanced Cell Diagnostics). Thereafter a serial amplification with different amplifying solutions (AMP1, AMP2, AMP3 and AMP4: alternating 15 min and 30 min at 40 °C) was performed. Between each incubation step, slides were washed with washing buffer. They were subsequently incubated with AMP 5, AMP 6 and DAB at RT for 45 min (AMP 5) and 15 min, respectively. Gill’s hematoxylin served to counterstain the sections which were then dehydrated with graded alcohol and xylene and coverslipped.

The following negative controls were included: a consecutive section incubated accordingly but without including the hybridization step, and sections from a cattle tested negative for BoHV-6 incubated according to the above protocol (Supplemental Figure S2).

## 3. Results

### 3.1. Histology

The 10 examined cattle, selected based on the BoHV-6 copy numbers in the tissue samples, comprised three necropsied animals from Switzerland (Nos. 1–3); these were aged 4.6 months (No. 3), 4.5 and 8 years (Nos. 2 and 1, respectively) and diagnosed with severe urachitis and periorchitis with abscess formation, pyonecrotic mastitis and serofibrinous peritonitis, respectively (Table 1). The remaining animals (Nos. 4–10) were healthy cattle slaughtered in the UK and aged between 13.2 and 33.6 months (Table 1). The histological examination of the lung, BLN, spleen and tongue specimens did not reveal any evidence of pathological processes in the selected animals. The histological changes did not go beyond agonal or slaughter associated changes, i.e., diffuse hyperemia of lung and spleen, consistent with acute congestion.

### 3.2. Target Cells of BoHV-6

**Figure 1:**
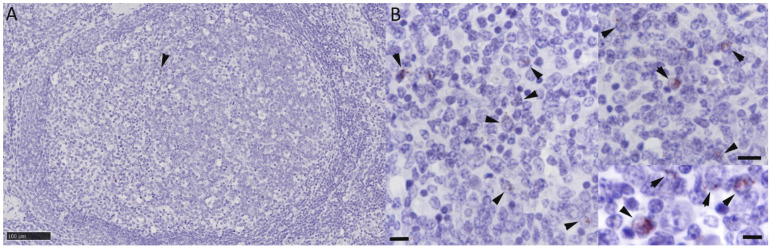
RNAscope^®^ RNA-ISH for BoHV-6 ORF73. Bronchial lymph node, animal No. 10. (**A**) Large secondary follicle with rare positive lymphocytes (arrowhead) in germinal center. Bar = 100 µm. (**B**) Higher magnification of germinal center with several lymphocytes exhibiting a positive signal of variable intensity (arrowheads). Bars = 20 µm (**left** and **top right**) and 10 µm (**bottom right**). RNA-ISH: hematoxylin counterstain.

Overall, the RNA-ISH resulted in very limited signal detection. In two animals (Nos. 3 and 5), it yielded no signal at all. In all remaining animals, signals were detected in the BLN. However, these were limited to nuclear signals in scattered lymphocytes in lymphatic follicles (Figure 1A,B).

**Figure 2:**
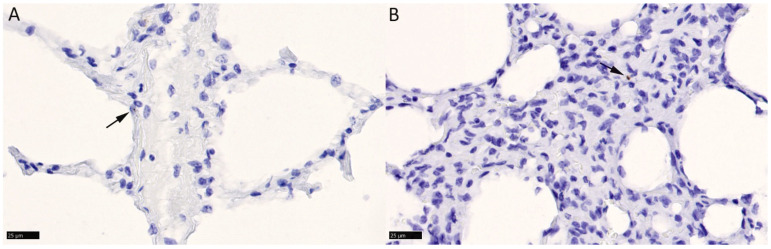
RNAscope^®^ RNA-ISH for BoHV-6 ORF73. Lung. (**A**) Animal No. 1. Alveoli with one pneumocyte exhibiting a nuclear signal (arrow). (**B**) Animal No. 8. Parenchyma with interstitial cell exhibiting a cytoplasmic signal (arrow). Bars = 25 µm. RNA-ISH: hematoxylin counterstain.

Interestingly, higher viral copy numbers as detected by qPCR were not associated with higher numbers of positive lymphocytes. In the lungs, where the main potential target cells would be the respiratory and alveolar epithelial cells, a signal was generally not detected. However, in animals No. 1 and 8, very rare pneumocytes and, possibly, infiltrating lymphocytes exhibited a weak signal (Figure 2A,B).

No signal was detected in any of the samples from the spleens. The same applies to the tongue, where the squamous epithelial cells of the mucosa would have been the main potential target cells, despite the partly very high viral copy numbers detected by qPCR in both organs (Table 1).

## 4. Discussion

Gammaherpesviruses are associated with a range of diseases. Several of these are observed mainly in dead-end hosts; a good example is MCF in ruminants [25]. In their natural hosts, however, many gammaherpesviruses are generally apathogenic or induce only limited and transient clinical signs before they settle in persistent, latent infections [26]; for some, reaction is a known feature [27]. It has not yet been fully elucidated whether gammaherpesvirus associated disease in the dead-end host is accompanied by or due to a shift in the viral target cell spectrum, although for macaviruses, the causative agents of MCF, there is evidence of this. Indeed, a shift from B cells to T cells is indicated in MCF, where viral protein and RNA is mainly detected in T cells in MCF lesions and also in the lymphatic tissue [23,24,28]. In sheep as the natural hosts of OvHV-2, experimental challenge leads to infection of lower airway epithelial cells, followed by persistent latent infection [17,29,30]. Also, experimental studies with MHV-68 in its natural host, the wood mouse, have shown that the virus infects pneumocytes early after infection, inducing a limited granulomatous inflammatory response and the formation of bronchus-associated lymphatic tissue (BALT); the virus then becomes latent in lymphocytes in BALT and other lymphatic tissues. In contrast, in laboratory mice, infection induces T cell-dominated pneumonia without BALT formation but latency in other lymphatic tissues [16].

A previous study of our group detected BoHV-6 in lung, lingual mucosa, BLN and spleen of infected cattle [4], which prompted the current in situ study to identify the viral target cells by RNA-ISH for viral ORF73 [3,5,29]. ORF73 mRNA is produced both during productive viral replication and, at low levels, during latency [2,31]. The RNA-ISH confirmed ORF73 transcription; however, this was very limited and only confirmed in lung and BLN, in very rare pneumocytes and interstitial cells in the former, and a few lymphocytes in the germinal centers in the latter. This discrepancy raises the question of sensitivity of the two methods. The applied qPCR is highly sensitive and allowed for the detection and quantification of very low infection levels, down to 1 viral copy/100 ng DNA [4]. In a recent study, we took a similar approach and applied RNA-ISH using the RNAscope^®^ ISH method to detect OvHV-2 target cells in PCR-positive tissue in a goat with MCF. We were able to detect Ov2.5 mRNA signals in a few individual keratinocytes in the lesioned skin, where the qPCR yielded >37,000 viral copies per 100 ng DNA [28]. Ov2.5, encoding viral IL-10, is expressed during both viral phases [22,32], similar to BoHV-6 ORF73. The tissues examined in the present study harbored BoHV-6 with a maximum copy number of approximately 20,000/100 ng DNA; it is therefore possible that the sensitivity of the RNA-ISH was too low to detect a signal in some cells.

BoHV-6 ORF73 transcription was observed in a few lymphocytes in the germinal centers, most likely B cells, in the BLN. This indicates that the virus infects and is latent in B cells, similar to MHV-68 in wood mice and EBV in humans as their natural hosts [16,33]. A signal was also observed in rare pneumocytes. In this respect, BoHV-6 appears similar to MHV-68 and OvHV-2 in their natural hosts [16,17]. Whether BoHV-6 targets the same cells in the spleen as in the BLN could not be confirmed; for MHV-68, this has been reported [16]. Based on the PCR results of our previous study, we hypothesized that BoHV-6 might shed orally, leading to contact transmission, like most herpesviruses [4]. However, with the current approach, we could not confirm ORF73 transcription in the oral mucosa, and, more specifically, the squamous epithelium of the tongue. The fact that OvHV-2-infected healthy lambs can shed infectious virus not only through nasal secretions, but also via the alimentary tract [34,35,36] indicate a similar target cell spectrum also with natural infection; however, the studies did not include in situ approaches to identify the viral host cells, different from another study that investigated OvHV-2 infected cattle by RNA-ISH and found evidence of viral transcription in the epithelium of the oral mucosa [23]. The present study did not investigate whether infected cattle carried BoHV-6 in the blood. However, this was shown in a previous study [6] and is similar to OvHV-2 in its natural host: in sheep, white blood cells are the first target of OvHV-2 and also mediate persistent latent infection [30,35,37,38]. So far, it has not yet been determined which blood leukocytes harbor BoHV-6.

The histological examination of the PCR-positive tissues did not reveal any pathological changes, which provides further evidence that BoHV-6 infection has no obvious consequence to the host, supporting that BoHV-6 can indeed be a true commensal in cattle.

## 5. Conclusions

In conclusion, the present study shows that BoHV-6 has a basic target cell spectrum similar to that of MHV-68, EBV and/or OvHV-2, in their natural hosts. We were not able to confirm that BoHV-6 is also harbored in the squamous epithelium of the oral mucosa and might indeed be shed from there, allowing for contact transmission. However, it provides further evidence that BoHV-6 is primarily an apathogenic commensal in cattle.

## Figures and Tables

**Table 1:** Animals included in this study. BoHV-6 copy numbers and results of RNA-ISH for BoHV-6 ORF73 in the examined tissues.

Animal No.	Main Diagnosis	Age (y)	Sex	Lung		BLN		Spleen		Tongue	
				Copy No	ISH	Copy No	ISH	Copy No	ISH	Copy No	ISH
1 ^A^	Severe serofibrinous peritonitis	8	F	4260	+	21,593	*	17,905	−	399	−
2 ^A^	Severe pyonecrotic mastitis	4.5	F	135	−	2541	+	8547	−	133	−
3 ^A^	Severe urachitis with abscess formation, periorchitis	0.4	M	4557	−	1482	−	5006	−	38	−
4 ^B^	None (healthy, slaughtered)	1.1	F	17	−	5302	+	268	−	81	−
5 ^B^		1.3	M	84	−	10	−	9668	−	3812	−
6 ^B^		1.8	F	93	−	1932	+	2498	−	1353	−
7 ^B^		2.3	F	238	−	3331	+	1161	−	0	−
8 ^B^		2.8	M	1371	+	2123	+	810	−	20,441	−
9 ^B^		2.3	M	570	−	2866	+	2142	−	8340	−
10 ^B^		1.75	M	1098	−	20,794	+	1099	−	973	−

Legend: ^A^ Switzerland; ^B^ UK; * RNA-ISH not performed due to poor tissue morphology; Copy Nr: BoHV-6 DNA copy number/100 ng DNA; +/−: (no) RNA-ISH signal detected.

## Data Availability

The article contains all data relevant to this study.

## Supplementary Materials

The following supporting information can be downloaded at: https://www.mdpi.com/article/10.3390/v16111730/s1, Figure S1: RNAscope^®^ RNA-ISH for Bos taurus peptidylprolyl isomerase B (PPIB); Figure S2: RNAscope^®^ RNA-ISH negative controls.
