# Supplementary material for: Bovine Gammaherpesvirus 6 Tropism in the Natural Host
Source: Viruses. 2024 Nov 3;16(11):1730. doi: 10.3390/v16111730 (PMC11598913; doi:10.3390/v16111730)
Supplement: Supplementary file 1 [file viruses-16-01730-s001.zip › viruses-3247109-supplementary.pdf]

Supplementary Materials

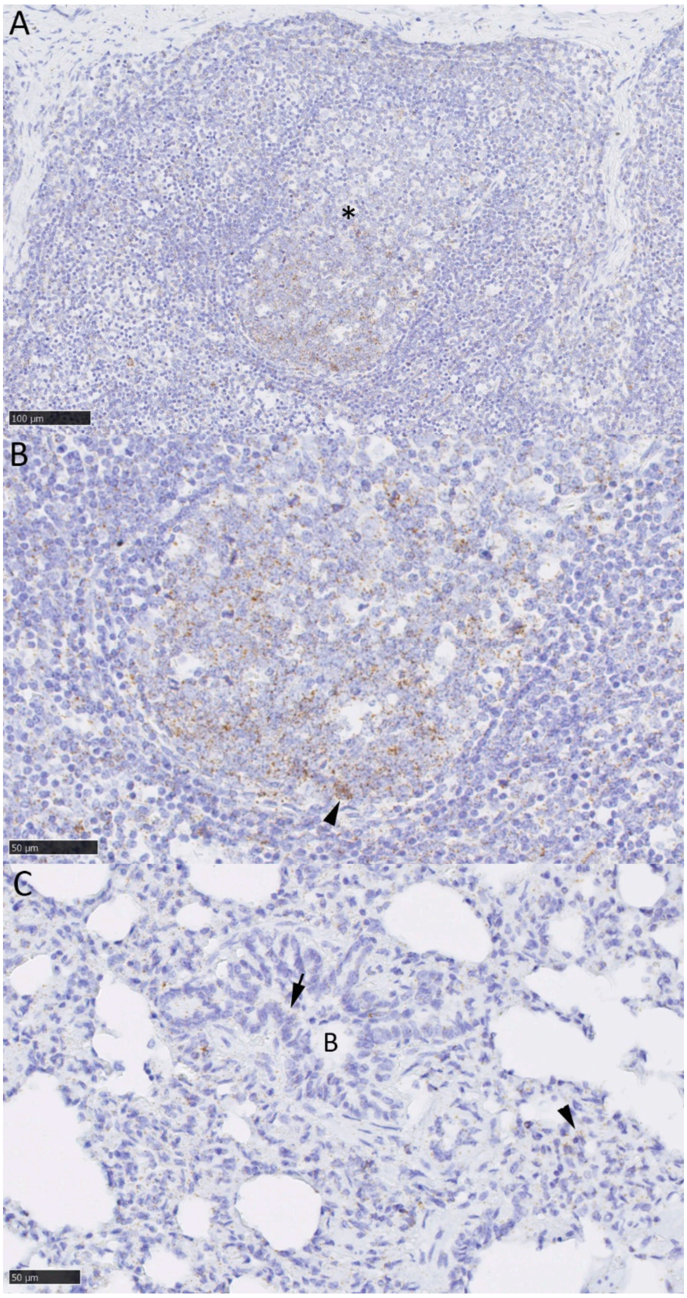

Figure S1. RNAscope® RNA-ISH for *Bos taurus* peptidylprolyl isomerase B (PPIB). Bronchial lymph node and lung, animal No. 10. The PPIB signal confirms RNA preservation and quality. **A)** Bronchial lymph node. The majority of cells within follicles (asterisk: germinal center) exhibit a weak to strong signal. **B)** Higher magnification of the follicle in A, highlighting the positive signal. The arrowheads points at a lymphocyte with a strong signal. **C)** Lung. Weak signals are seen in alveolar epithelial cells (arrowhead) and respiratory epithelial cells (arrow) in bronchioles (B). RNA-ISH, hematoxylin counterstain.

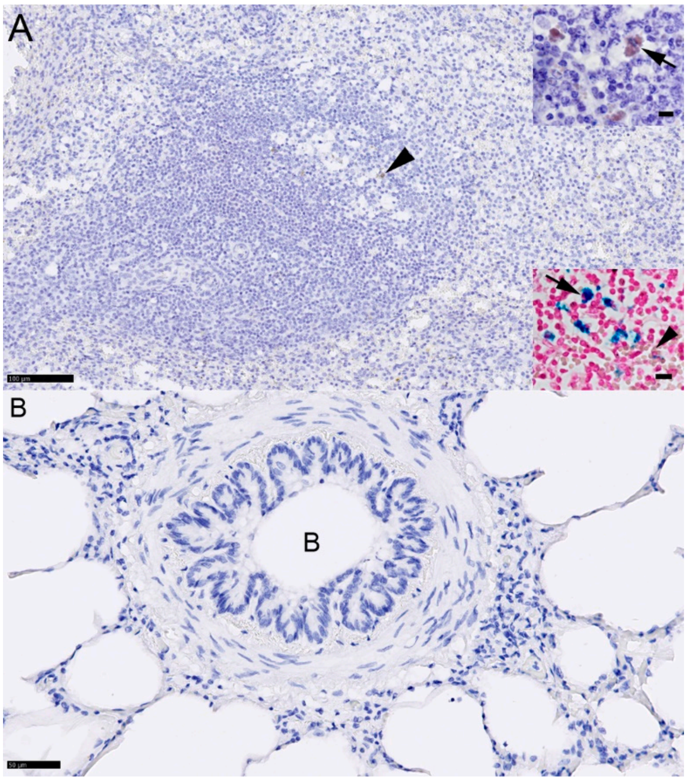

Figure S2. RNAscope® RNA-ISH negative controls. **A)** Spleen, BoHV-6 positive animals. Section incubated according to the protocol but without including the hybridisation step. Arrowhead: pigment laden cells in follicle centre. A higher magnification (top inset) shows the pigment laden macrophages (arrow). A consecutive section stained for iron (bottom inset; Prussian Blue stain for Fe^{3+}) confirms the pigment as hemosiderin (arrow; blue color) and also highlights macrophages that contain both iron and phagocytosed erythrocytes (arrowhead). **B)** Lung, BoHV-6 negative cattle (confirmed by PCR). There is no signal. B: bronchiole. RNA-ISH, hematoxylin counterstain.
